# Current status and priorities of paediatric oncology nursing in Africa: a synthesis of perspectives from SIOP Africa nurses

**DOI:** 10.3332/ecancer.2023.1585

**Published:** 2023-08-10

**Authors:** Glenn M Afungchwi, Elianeth Kiteni, Mariam Ndagire, Biemba Maliti, Rachael Kunkel, Julia M Challinor, Rachel Hollis

**Affiliations:** 1 The University of Bamenda, Bamenda, Cameroon; 2 Muhimbili National Hospital, Dar es Salaam, Tanzania; 3 Uganda Cancer Institute, Kampala, Uganda; 4 Zambia Cancer Diseases Hospital, Lusaka, Zambia; 5 Arkansas Children's Hospital, Little Rock, AR 72202, USA; 6 University of California San Francisco, 2 Koret Way, San Francisco, CA 94143, USA; 7 Leeds Children’s Hospital, Clarendon Wing, LS1 3EX Leeds, UK

**Keywords:** nursing status, Africa, global initiative, childhood cancer, paediatric oncology

## Abstract

**Introduction:**

As African countries adopt the global goal of improving childhood cancer survival to 60% by 2030, intentional actions are required to improve nursing. This report aims to describe the current status of paediatric oncology nursing in Africa.

**Methods:**

We report on nursing-related aspects of a survey to map paediatric oncology services in Africa (2018–2019), document perceived nursing strengths and weaknesses (2017) and share nurses’ research priorities (2019). Additionally, we report on a survey to identify topics for a foundation course (2019) and the expressed perspective of African nurses about the status of paediatric oncology nursing across the continent (2022).

**Results:**

Only 21% of respondents in the African mapping survey reported having nurses who care for children with cancer at least 75% of the time. Many centres do not have allied health workers like dieticians and play therapists, thus contributing to the nursing burden of care. The main strength of African paediatric oncology nurses was the humanisation of care, while the major weakness was the lack of training follow-up. The top research priorities focused on professional practice and psychosocial support. The Delphi survey identified 57 topic areas grouped into a 12-module curriculum for nurses new to paediatric oncology. The nurses affirmed their dedication to providing compassionate care, however, noted their vulnerability to harm and called for better specialisation, recognition and remuneration.

**Conclusion:**

This paper amplifies the voice of African paediatric oncology nurses. It illuminates the room for improvement and provides a reference point for future comparison.

## Introduction

In 2020, the World Health Organisation (WHO), the International Council of Nursing and Nursing NOW published the first ‘State of the World’s Nursing Report’ [1]. This was followed in 2021, by the World Health Assembly endorsement of the WHO's Global Strategic Directions for Nursing and Midwifery, which focused on four policy areas: nursing education, jobs, leadership and service delivery; all are pertinent themes for paediatric oncology nurses across Africa [2].

In 2022, the International Society of Paediatric Oncology (SIOP) Africa Continental Congress provided the opportunity for paediatric oncology nurses from across the region to meet for the first time since 2019. This Congress has become an opportunity for the African paediatric oncology nursing community to network, collaborate and present a shared voice. It provided the opportunity to reflect on significant global and regional developments in children’s cancer care and the world of nursing.

In 2018, the WHO announced the start of the global initiative for childhood cancer to save more than one million lives and improve survival to 60% worldwide by 2030 [3]. According to Denburg *et al* [4], ‘National governments must be convinced of the potential for foundational health system strengthening through attention to childhood cancer care, and the presence and capability of networked actors primed to amplify public sector investments and catalyze change on the ground’. The WHO global initiative for childhood cancer currently includes three focus countries in the African region: Ghana, Morocco, and Zambia, with Zimbabwe, Senegal and Cameroon at earlier stages of inclusion [5]. Ministries of health have engaged with WHO and local stakeholders, including non-governmental organisations, parent groups, and hospitals providing childhood cancer care, to perform situational analyses and create strategic plans for strengthening early diagnosis, access to care, sustainable essential medication supply and improved survivorship. None of these goals is possible without competent and reasonably resourced nursing care and a sustainable nursing workforce [6].

In July 2019, the African Union held a cancer symposium with the Organisation of African First Ladies for Development in Niger to bring awareness to the increasing cancer burden on the continent and the need for advocacy, financing, and resource development (including workforce resources) at an international, regional and local level [7]. African nurses are critical partners in this effort since they are the single largest workforce, have deep community ties, and, when specialised, have the knowledge and skills to provide the care required for patients with cancer in their region, including children and adolescents. Nursing oncology specialisation was supported by a call in 2017, when the WHO passed resolution 70.12, ‘Cancer prevention and control in the context of an integrated approach,’ recognising cancer as a ‘…growing public health concern’ and urging member states to ‘…facilitate cross-sectoral cooperation between health professionals, as well as the training of personnel at all levels of health systems…’ among other cancer control measures [8, p 4].

With the current global momentum in the improvement of childhood cancer and the status of nursing, it is essential to document the baseline situation of paediatric oncology nursing in Africa. Our aim is, therefore, to describe the current status of paediatric oncology nursing in Africa, a continent with a population of 1.4 billion [9], as understood and described by the nurses themselves in the meetings of the SIOP Africa and through their scholarly activities over the last 5 years.

## Method

**Figure 1.**
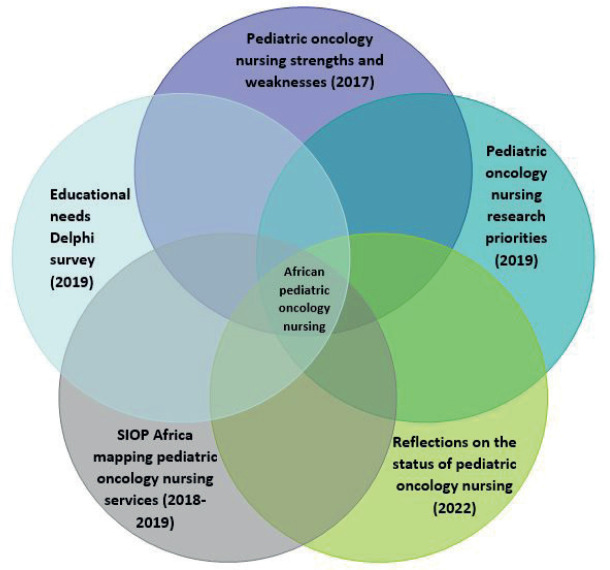
Venn diagram of five data sources presented to illustrate the status of African paediatric oncology nursing (2017–2022).

For this article, we have used five distinct but overlapping data sources from African paediatric oncology nurses’ contributions at professional conferences, research and survey responses in the context of paediatric oncology nursing in Africa (Figure 1).

We begin with the results of a SIOP Africa mapping exercise (2018–2019) that documented childhood cancer services, including nursing workforce and nursing practice-related data. We go on to synthesise the perspective of frontline paediatric oncology nurses across Africa obtained at multiple meetings in an orderly manner, using distinct surveys. We first report on Francophone and Anglophone African paediatric oncology nurses’ self-identified strengths and weaknesses as documented at the 12th SIOP Africa Congress in 2017. We share African paediatric oncology nurses’ priority topics for education and training obtained through a Delphi survey conducted in 2019, and priority topics for nursing research identified at the 13th SIOP Africa Congress in 2019. Finally, we include the findings of a survey question on the priorities for paediatric oncology nursing distributed to all nurse attendees at the 14th SIOP Africa Congress in 2022. This report intentionally does not follow a strict chronological order as we first present the global mapping data to set the stage with available paediatric oncology nursing services before discussing specific aspects of these services. A discussion at the end, supported by published literature, articulates the current status and priorities for paediatric oncology nursing in Africa (Figure 1).

In 2018, SIOP conducted a mapping exercise by online survey to identify global childhood cancer services, beginning in Africa [10]. The only nurse-specific data were the number of nurses working 75% of their time in paediatric oncology units. However, to demonstrate the burden of care for these nurses, data on the availability of allied health professional support, e.g., psychologist/psychiatrist, dietician/nutritionist, physiotherapist, social worker, pharmacist and palliative care team were also extracted from survey responses. Additionally, data regarding spiritual/religious, volunteer and patient/family support groups were extracted as well as the presence of dedicated paediatric oncology units and access to paediatric intensive care units (ICU) to provide the context of the nursing work environment and family support.

In March 2017, a workshop at the 12th Annual SIOP Africa Continental Conference in Marrakech, Morocco convened 50 nurses from Francophone and Anglophone Africa to identify strengths and weaknesses of the paediatric oncology nursing workforce in their settings, guided by a projected slide with a table of Strengths/Weaknesses/Opportunities/Threats in English and French for analysis [11]. Two bilingual moderators led the discussion as the nurses’ oral answers were documented on a flip board for the audience to consider. Suggestions supported by >80% of participants were documented. The rich and detailed discussion of strengths and weaknesses, with oral translation of each nurse's contribution, as well as the time allotted for this exercise in the conference programme did not allow for the exploration of threats and opportunities as had been anticipated.

In March 2019, at the 13th SIOP Africa Continental conference in Cairo, Egypt, 30 nurses from Egypt, Tanzania, Morocco, Uganda and Cameroon participated in a 2-day workshop to identify research priorities for paediatric oncology nurses in Africa. Participants formed small groups and submitted research questions to share with the larger group. Submissions were collected, collated and duplicates removed. Responses were thematically coded using Atlas.ti version 8. Themes were grouped, first by research priorities, and second, by target population.

At the SIOP Africa, Cairo Conference, the nurse attendees also met to initiate a project to identify the educational priorities of sub-Saharan nurses caring for children/adolescents with cancer. Following the conference, an on-line Delphi Survey in English and French using REDCap was distributed to a convenience sample of nurses from sub-Saharan Africa with experience of at least 1 year caring for children with cancer [12]. The sample was recruited with a snowball methodology using existing networks of childhood cancer nurses in both Anglophone and Francophone Africa as well as an extensive WhatsApp group of 172 members. In the first round, participants were asked to identify a minimum of 10 and a maximum of 20 topic areas for inclusion in a ‘foundation’ level programme for paediatric oncology nurses. In the second round, participants were asked to rate the importance of each topic identified on a 5-point Likert scale with an aim of 80% consensus on topics to be addressed. In round three, participants were presented with a proposed outline framework of a nursing curriculum and asked to express agreement with the wording and grouping of topics into modules to develop an outline curriculum framework.

In 2022, at the 14th SIOP Africa Continental conference in Kampala, Uganda, approximately 50 nurses gathered once again from Francophone and Anglophone countries. The nurses were asked to write one sentence in English about their priority for paediatric oncology nursing in Africa using an anonymous Google form. Comments were collected on a Google form and inductive thematic analysis was conducted using Atlas.ti 9.

## Results

### 2018-2019 SIOP global mapping Africa

In the initial effort of the SIOP Global Mapping Programme survey, a total of 109 responses were received from facilities across 46 African countries [10]. Information about facilities’ physical infrastructure for paediatric oncology is detailed in Table 1. Only nine responses were from nurses (8.3%); the rest were primarily from physicians: heads of division (*n* = 47, 43.5%), consultants (*n* = 46, 41.7%), and junior faculty (*n* = 3, 2.8%). There was one survey question about how many nurses care for children with cancer at least 75% of the time. Twenty-three participants (21%) reported that they had no nurse who cared for children with cancer >75% of the time at their centres, 30 (28%) had 1–5 nurses who did so, 20 (18%) had 6–10, and 6 (5%) had 11–15. Ten (9%) of centres had 16–20 nurses caring for children >75% of the time, while seven (6%) had >20 nurses who did so. For every unit increase in the number of beds, there was a 10% reduction in the number of nurses working for more than 75% of their time in paediatric oncology. This was, however, not statistically significant (*p* = 0.313).

Greater than two-thirds (66%) of the facilities had a dietician/nutritionist, physiotherapist, social worker, or pharmacist. Approximately half (53%) had volunteers, a palliative care team, a psychologist, spiritual/religious support, and patient support groups. One-third or less (28%) had a play therapist/child life specialist, bereavement counsellor or schoolteacher (Table 2). However, the survey did not ask if these professionals were dedicated only to caring for children with cancer.

### SIOP Africa 2017: strengths and weaknesses of paediatric oncology nursing in Africa

The paediatric oncology nurses identified the humanisation of care, ‘making the patients feel welcome when arriving and staying at the hospital for childhood cancer treatment’ as a strength. They emphasised being close to their patients and sharing their patients’ emotions and feelings during their treatment period. They said they focus on delivering safe care with the patience and courage to face a challenging patient population. The nurses identified their strengths in pain assessment and control while caring for children and adolescents with cancer using validated assessment tools. They believed they were strong in explaining cancer care and treatment to their patients and families. Finally, they described their competence and mastery of nursing practice (professional competency) for this patient population, as well as their desire to improve.

The nurses identified that paediatric oncology nurses in their settings in Africa were weak in re-evaluating nursing training to determine if there had been any impact on patient care. Participants believed that reassessing practice after beginning work with children and adolescents with cancer was essential for providing high-quality nursing care. Second, the nurses stated they were weak in knowledge and skills to conduct research and submit manuscripts for publishing. They further noted a severe lack of evidence about nursing practice in their various settings across Africa. Poor documentation of nursing actions was identified as a weakness. The nurses believed this impacted patient care and was an issue that needed to be addressed across Africa. Participants all stated that the shortage of trained nursing staff was a major cause of their weaknesses. A final weakness identified in this workshop was the lack of knowledge and skills to conduct research and submit manuscripts for publishing.

### SIOP Africa 2019: research priorities for paediatric oncology nursing in Africa

**Figure 2.**
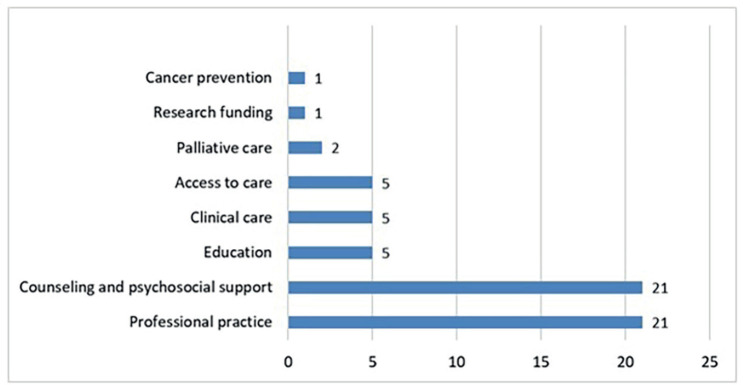
Priority research topics for paediatric oncology nursing in Africa as identified by nurses participating in the 2019 13th SIOP Africa Congress, in Cairo, Egypt.

**Figure 3.**
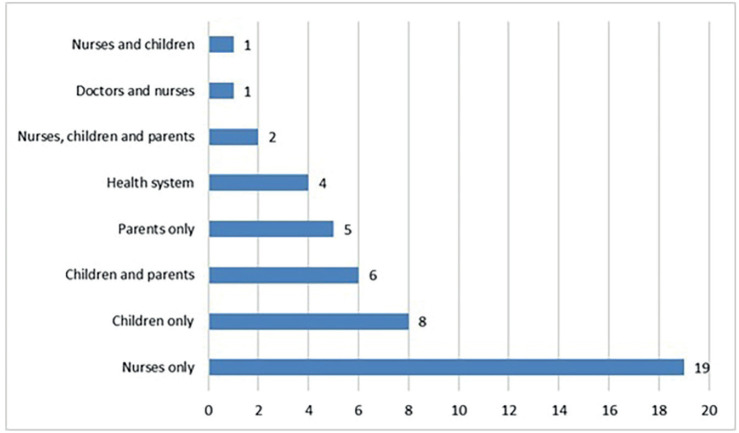
Research targets for paediatric oncology nursing in Africa as identified by nurses participating in the 2019 13th SIOP Africa Congress, in Cairo, Egypt.

The nurse participants at SIOP Africa 2019 in Cairo, Egypt, submitted 54 research questions; 46 were retained once duplicates had been removed. Eight main themes for research were identified, with the two most common being professional practice and counselling and psychosocial support (Figure 2). The principal research population priority was nurses, alongside parents and children (Figure 3).

### Post-SIOP African conference 2019: educational priorities for nurses new to paediatric oncology nursing in Africa

**Figure 4.**
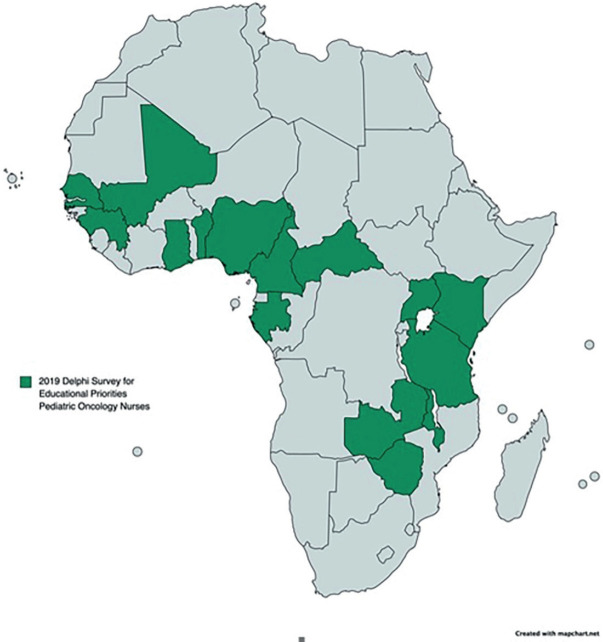
Countries of the paediatric oncology nurses participating in the educational priorities for paediatric oncology nurses Delphi survey for sub-Saharan Africa, SIOP Africa meeting 2019, in Cairo, Egypt.

In the Delphi survey to identify topics essential for a foundation training programme, 46 nurses responded in the first round and 57 topic areas were identified in the 5 most common included a general introduction to cancer and treatment modalities, chemotherapy administration and side effects, tumour lysis syndrome, palliative care, and infection prevention and control (Figure 4). Twenty-eight (63%) of round one participants responded to the second round of the survey. All topic areas except one achieved consensus of 80%. Fifty-six topics were identified with several overlap. Topics were grouped to 12 modules (Table 3). The 12 proposed modules were sent to all participants from rounds one and two. Minor modifications were made to the wording of the topics. Nineteen (41%) participants commented on 12 modules encompassing all topics. There was 98% agreement for wording and 97% for grouping.

### SIOP Africa Congress, Kampala, Uganda 2022: paediatric oncology nursing in Africa

Forty-five participants responded to the survey distributed during the 2022 SIOP Africa Congress, providing one sentence each giving their perspective on paediatric oncology nursing in Africa. Four major themes were identified, including the general nature of paediatric oncology nursing and nurses in Africa, training and certification, contribution to improved survival, and the need for more recognition and support.

### Theme 1: General nature of paediatric oncology nursing and nurses in Africa

Paediatric oncology nursing was defined as a practice that provides professional holistic care as one participant put it:

‘*Pediatric oncology nursing is a specialized discipline that requires knowledge about pediatric oncology care, passionate, empathetic, loving, caring and hard-working. It entails holistic care, including psychosocial, emotional, physical and spiritual. The nurse must do their best and hope for the best. All in all, it is not ordinary nursing, so both the nurse and the patient require psychological care.’* **Participant 23**

The nurses reported a high level of commitment and motivation to meeting the care needs of their patients.

‘*The most dedicated and caring people full of compassion for the little souls in their hands.’* **Participant 26**

‘*They are determined to serve despite limited resources.’*

However, the nurses lament their vulnerability to harm and discouragement in their practice and advocate for more attention to their occupational safety needs and support for their emotional well-being. **Participant 29**

*‘We are giving it our all and never giving up on our kids; thus, we need all the support out there for the job.’* **Participant 7**

*‘Nurses in Africa will love to see more protective wear/equipment that can be used during chemotherapy to help protect them from too much exposure.’* **Participant 32**

### Theme 2: Training and certification

The nurses acknowledge the availability of trained colleagues

*‘We have qualified and competent pediatric oncology nurses in Africa.’* **Participant 45**

They, however, express the need for more specialised training with certification for better outcomes.

‘*To build the capacity of specialists in pediatric oncology nursing in Africa so as to improve the cure rates for children with cancer.’* **Participant 36**

‘*Pediatric oncology nurses need training and certification so that they can be recognized worldwide.’* **Participant 1**

### Theme 3: Contribution to improved survival and palliative care

The nurses believe that despite the limited resources for care at their disposal, they have contributed significantly to saving the lives of children with cancer and are to be relied upon for better survival. They also mention their essential role in palliative care.

*‘African nurses are the driving force for pediatric oncology in Africa. Investing in nurses will help Africa quickly reach the 60% by 2030 [(WHO) Global Initiative for Childhood Cancer (GICC)].’* **Participant 4**

*‘All pediatric oncology nurses should be introduced to pediatric palliative care because it makes a whole positive difference for the patients and their families.’* **Participant 18**

### Theme 4: Need for more recognition and support

The nurses believe that they are not adequately remunerated and call for more recognition of their services and resources to support training and education.

*‘Pediatric oncology nursing in Africa is emotionally draining; therefore, the nurses committed to work in this department need incentives to encourage them to keep at it.’* **Participant 11**

*‘African nurses need the training in pediatric oncology nursing to better give quality nursing care. They also need to be members of organizations like SIOP, so they are updated regularly on new things. SIOP needs to have a deliberate policy where membership from Africa is exempted from paying membership fees so that they do not pull out. African countries need financial help to be conducting orientation to new nurses in pediatric oncology so as to meet the baseline standards.’* **Participant 34**

## Discussion

The WHO GICC is building momentum within the specialty of paediatric oncology nursing. This paper provides the perspective and amplifies the voice of African paediatric oncology nurses and their advocacy to further the aim of prioritising services for children with cancer at national, regional and international levels.

The SIOP Global Mapping Programme survey of childhood cancer services in Africa provided the first 'bird's eye view' of where services exist and paediatric oncology nurses practice, often in highly challenging circumstances with limited resources [10]. Results demonstrated marked disparities between countries on the continent, where some have highly specialised services while others have none [10]. In many African settings, nurses take on roles otherwise undertaken by allied health and social care professionals in better-resourced settings.

For paediatric oncology nurses to deliver quality nursing care to their patients, they require systems and programmes developed to support this. However, more than 74.6% of the centres across Africa had only basic services with very few state-of-the-art services. Sirohi *et al* [13] note, Initiatives are being undertaken in low-and middle-income countries (LMICs) to deliver optimal cancer care by developing cancer centres, but many of these initiatives are currently fragmented and uncoordinated. The challenges of quality, value, affordability, and equality that cancer centres in high-income countries (HICs) face are multiplied in LMICs.

As noted in a study from Tanzania, to improve paediatric cancer outcomes, there is the need to strengthen training and diagnostic capacities, develop cancer registries and establish research databases in Africa [14]. These efforts should include nurse training and research to develop best practices in an African setting to be most effective.

The data provided by nurses themselves were limited, echoing the general paucity of publications on the experiences and aspirations of African paediatric oncology nurses. This is compounded by barriers including limited access to training and education, lack of professional resources and the hurdles faced to achieve publication of their work. This is further evidenced by a literature review of paediatric oncology nursing research in LMICs 2008–2018, which found only six publications from Africa [15].

The most fundamental and essential concepts in paediatric oncology nursing are compassion and compassionate care. Pehlivan and Güner [16] argue that ‘compassionate behaviour requires understanding others’ value, establishing a relationship with them, and responding in a way that is meaningful for that person’. A patient, parent and healthcare provider empirical model demonstrates four key domains in paediatric oncology compassion care (beneficence, human relating, seeking to understand, and attending to needs) [17]. The four domains align with those which nurses described as their strengths during the 2017 SIOP Africa Congress (humanisation of care for children, empathy for the patients during their treatment period, pain assessment and control using validated tools and good communication skills) despite the challenges they face in their hospital settings. Sedaghati *et al* [18]. further report that the majority of oncology nurses have positive attitudes towards empathic behaviour with cancer patients.

Educational opportunities such as Project ECHO (Extension for Community Healthcare Outcomes) seminars for nurses in sub-Saharan Africa, and nurse training activities within the Francophone Africa Paediatric Oncology Group and the collaborative African network for childhood cancer care and research have enhanced access to expertise in the care of children with cancer and contributed to enhancing paediatric oncology nurses’ knowledge and skills in Africa [19–21]. Good communication skills during cancer care and treatment are an important aspect of nursing children and adolescents with cancer and their families; the nurses believed this was also their strong point. For example, Graetz *et al* [22, p 4] found that ‘nurses, rather than psychosocial providers, provide most of the counselling and are available to clarify information discussed by the physician’ in Uganda.

Despite the limited exposure to and experience in research, nurses participating in the 2019 13th SIOP Africa Congress research priorities exercise showed a desire to base their nursing practice on evidence. In order to build that evidence base, nurses require further training, mentoring and funding to engage in nursing research and generate appropriate evidence for nursing practice in consideration of local, resource-limited practice environments. From the identified thematic areas, the two commonest research areas identified were issues surrounding professional practice and counselling/psychosocial support for patients and families. Paediatric oncology nursing practice is culturally and context-driven and requires locally acquired evidence to support best practices [23]. African nurses have a key role to play in current and future clinical trials, as evidenced in HICs [24], but will require capacity building to be able to do so [25]. Access to research training, funding and protected time are key steps to guide and motivate clinical nurses as well as academic nurses to participate in nursing and multidisciplinary research [23].

The SIOP baseline nursing standards recommend that paediatric oncology nurses should not rotate in other service units [26]. However, from the African mapping survey, only 23% of facilities had fixed nurse staffing in paediatric oncology units. The rotation of experienced paediatric oncology nurses away from oncology units means taking away the expertise, knowledge and experience that is integral to successful mentorship and quality care. Rotation, therefore, compromises the quality of care given to paediatric oncology patients and can also be stressful for the nurses. The nature of cancer and the treatment process itself often leaves the children acutely unwell, requiring critical care in ICU. About 38.0% of the African facilities had no paediatric ICU available; unfortunately, the few facilities with an ICU had limited equipment and personnel with paediatric oncology experience. Only one-fifth of the facilities had a paediatric ICU with all necessary equipment and personnel with paediatric intensive care expertise. According to Zinter *et al* [27, p 1536] ‘up to 38% of children with cancer require paediatric intensive care unit (PICU) admission within 3 years of a cancer diagnosis, (even in HIC), with reported PICU mortality of 13%–27% far exceeding that of the general PICU population’. The lack of ICU services or specialised staff across Africa may explain the causes of some preventable deaths among children with cancer.

The need for competent paediatric oncology nurses is one aspect of service provision that is universally acknowledged as essential to all strategies designed to improve access to care [26]. Competencies are fundamental to quality nursing practice and involve the application of critical thinking, knowledge, technical, and interpersonal skills demonstrated in the performance of skills in a defined context [28]. The development of training programmes for specialisation in paediatric oncology nursing on the African continent cannot be overemphasised in order to have competent specialists leading to better outcomes to care as was reinforced in the recent lancet oncology commission on cancer in sub-Saharan Africa [28]. Having sufficient theoretical, and clinical experience and attitude can also be significant in improving the morale of the nurses [29]. The 2019 Delphi survey following the 2019 SIOP Congress in Cairo, Egypt, further underscores the importance of training as revealed by the consensus on priority areas of education vital for nurses new to paediatric oncology nursing. The finding of this survey was condensed into a 12-module curriculum framework. This framework was piloted in Ghana in November 2021 and lead facilitators were trained in Malawi, in March 2022 [30].

A good rapport between the nurse and family enhances the nurse's ability to tailor their care to each individual child and helps the nurse to know what small things they could do for the child that would make a big difference [31]. During the 2022 SIOP Africa Congress in Kampala, Uganda, a survey regarding impressions of nurses about paediatric oncology nursing in Africa demonstrated the holistic nature of nursing care for children with cancer and high level of commitment and motivation from nurses to meeting the care needs of their patients. Nukpezah *et al* [29, p 7] speaking about paediatric oncology nursing in Ghana stated that ‘…pediatric oncology nurses mustn’t become task orientated… and lose sight of the holistic and human aspects of pediatric oncology nursing caring practice’.

Successful and effective paediatric cancer care and treatment requires quality care delivered by skilled professional nurses. Professional requirements include not only specialisation but psychological support and motivation to uphold an unwavering commitment to meeting the needs children with cancer and their families, and thereby contributing positively to better survival as espoused in the WHO *CureALL* framework of the GICC initiative [32]. The need for the expansion of educational programmes, the ‘…development of evidence-based practices for health promotion and well-being…’ as well as ‘…guidance to enhance and standardize the nursing care of children with cancer…’ have been recognised as key initiatives for improving paediatric oncology nursing [33, p 162]. The requirement for specialised training with certification was echoed again by SIOP Africa nurses to improve the numbers of available qualified and competent paediatric oncology nurses. However, the high demands of paediatric oncology nursing in resource-limited settings are not appropriately recognised or remunerated by health authorities such as hospital administrators and ministries of health. This can be improved by encouraging membership of relevant professional bodies such as SIOP that engender growth and development in clinical, educational, leadership and research; areas that have already been alluded to as requiring investment and growth.

## Limitations

While we use the information gathered through the reported surveys to build a picture of the status of paediatric oncology nursing in Africa, we recognise a number of limitations to this report. All surveys were conducted with a convenient sample of nurses who attended SIOP Africa conferences and therefore, were not a comprehensive representative sample. While the Delphi survey was distributed using a snowball technique, only colleagues whose contacts were available to the research team had the opportunity to take the survey or share it. The SIOP global mapping survey had very few nurse respondents and information about the one nursing data point was mostly provided by physicians. Additionally, due to the number of nursing research activities reported here, details on the methodology of each study are limited.

## Conclusion

This paper has identified factors that influence paediatric oncology nursing practice across Africa. It has presented published and unpublished evidence from data gathered over the last 5 years through a range of activities led by African paediatric oncology nurses themselves. The work provides a baseline of the current situation, articulates professional priorities, and sets goals for moving forward to strengthen paediatric oncology nursing development and improve the care of children/adolescents with cancer and their families across the African continent. Next steps for developing paediatric oncology nursing in Africa include country-level quality improvement exercises specific to nursing care and formulation of action plans and dedicated working groups to tackle specific objectives. The WHO global initiative for childhood cancer, which currently has five African countries in various stages of participation, provides a golden opportunity to implement this nursing strategy and evaluate its impact on the survival and care of children and adolescents with cancer. This is currently being done in Zambia and should be applied across all focus countries of the initiative [34].

## Conflicts of interest

The authors have no conflicts of interest to declare.

## Funding

None of the authors received any funding for the surveys included nor for the preparation of this manuscript.

## Author contributions

Glenn Mbah Afungchwi contributed to conceptualising the study, data collection and analysis, writing the manuscript. Elianeth Kiteni contributed to conceptualising the study, data collection and analysis, writing the manuscript. Mariam Ndagire contributed to conceptualising the study, data collection and analysis, writing the manuscript. Biemba Maliti contributed to conceptualising the study, data collection and analysis, writing the manuscript. Rachael Kunkel contributed to conceptualising the study, data collection and analysis, writing the manuscript. Julia Challinor contributed to conceptualising the study, data collection and analysis, writing the manuscript. Rachel Hollis contributed to conceptualising the study, data collection and analysis, writing the manuscript.

## Figures and Tables

**Table 1.** Details of 109 paediatric oncology facilities reporting on the SIOP global mapping survey from 46 African countries.

Support services	Have resource	Do not have resource	Do not know
Pharmacist	91%	8%	1%
Social worker	81%	15%	4%
Physiotherapist	78%	18%	4%
Dietitian	66%	30%	4%
Volunteers	53%	44%	3%
Patient support group	49%	49%	2%
Psychologist	45%	51%	3%
Palliative care	43%	53%	4%
Spiritual/Religious	40%	53%	7%
School teacher	33%	63%	4%
Child life worker/Play specialist	28%	70%	2%
Bereavement	23%	70%	7%

**Table 2.** Presence of allied health workers in 109 facilities in 46 African countries responding to the SIOP Africa mapping survey 2018.

What type of dedicated paediatric oncology programme does your hospital have?	
Pilot projects	10.1%
Some basic oncology	24.2%
Established oncology programme with most basic services and a few state-of-the-art services	40.4%
Paediatric oncology programme with all essential services and most state-of-the-art services	14.1%
State-of-the-art services and some highly specialised services (e.g., proton beam radiation therapy, MIBG therapy, phase I studies)	3.0%
Don’t know	8.1%
Dedicated paediatric oncology ward?	
No paediatric oncology inpatient ward	19.2%
Area of the hospital where children with cancer are admitted when possible; frequent overflow to other wards; no fixed staff	14.1%
Paediatric oncology inpatient ward available to most patients with limited, fixed staff (e.g., oncology nurse permanently assigned)	23.2%
Paediatric oncology inpatient ward separate from inpatient units for other patients; sufficient beds such that oncology patients rarely require admission to other wards	34.3%
Subspecialised paediatric oncology wards (e.g., transplant, neuro-oncology, acute myeloid leukaemia)	8.1%
Don’t know	1.0%
Do children with cancer have access to paediatric intensive care facilities at your hospital?	
ICU present; limited equipment and personnel with limited paediatric experience	38%
Mechanical ventilators, inotropes, central venous access, dialysis; personnel with some paediatric experience	16%
Paediatric ICU with all necessary equipment and personnel with paediatric intensive care expertise	19%
Advanced cardiopulmonary support available (extracorporeal membrane oxygenation)	4%
No	21%
Don’t know	2.0%

**Table 3.** Final topic results of sub-Saharan Africa nurses network Delphi survey 2019.

**Module 1:** General introduction to cancer
**Module 2:** Diagnosis of cancer
**Module 3:** Cancer treatment modalities
**Module 4:** Management of chemotherapy side effects
**Module 5:** Paediatric oncological emergencies
**Module 6:** Care of the sick child
**Module 7:** Providing safe care
**Module 8:** Psychosocial care
**Module 9:** Nutritional support for children with cancer
**Module 10:** Palliative and supportive care to a child with cancer
**Module 11:** Outpatient and follow up care
**Module 12:** Paediatric oncology nursing and the role of the Nurse
